# Olfactory-Cleft Biopsy in Alzheimer’s Disease: An Emerging Neuroimmune Window into Preclinical Pathobiology

**DOI:** 10.3390/ijms27188043

**Published:** 2026-09-10

**Authors:** James Chmiel, Aleksandra Kładna

**Affiliations:** 1 Faculty of Physical Culture and Health, Institute of Physical Culture Sciences, University of Szczecin, Al. Piastów 40B Block 6, 71-065 Szczecin, Poland; 2 Department of the History of Medicine and Medical Ethics, Pomeranian Medical University, Ul. Rybacka 1, 70-204 Szczecin, Poland

**Keywords:** Alzheimer’s disease, olfactory cleft, olfactory epithelium, brush biopsy, preclinical Alzheimer’s disease, neuroimmune interface, single-cell RNA sequencing, olfactory sensory neurons, CD8 T cells, neuroinflammation, biomarker discovery, patient-derived cellular models

## Abstract

Olfactory dysfunction is an early non-cognitive feature of Alzheimer’s disease (AD), but smell impairment has traditionally been used mainly as a behavioral marker. This review examines the emerging use of the olfactory cleft as an accessible source of living neuronal, epithelial, progenitor, and immune cells for studying AD pathobiology. Histopathological and patient-derived culture studies have reported amyloid-β, tau, oxidative-stress, mitochondrial, biometal, and transcriptional abnormalities in olfactory tissue. More recent endoscopically guided brush sampling with single-cell profiling has identified activated memory CD8 T-cell states, inflammatory myeloid programs, and neuronal metabolic changes, including in cognitively unimpaired individuals with abnormal cerebrospinal-fluid amyloid biomarkers. These findings support olfactory-cleft sampling as a research platform for investigating early neural–immune changes, but current evidence is based on small, largely cross-sectional cohorts and does not establish disease specificity, causality, or prognostic utility. Longitudinal multicenter studies integrating olfactory-tissue profiling with established fluid, imaging, genetic, cognitive, and olfactory biomarkers are required before clinical translation.

## 1. Why Olfaction Matters in Alzheimer’s Disease

Olfactory dysfunction is one of the most consistent non-cognitive features of Alzheimer’s disease (AD), but it has traditionally been used as a clinical screening signal rather than as a direct route into disease biology [1,2]. This distinction is changing because the olfactory neuroepithelium can be sampled in living individuals and contains neuronal, epithelial, regenerative, and immune compartments that can be examined with modern molecular methods [3,4]. Its connections with early-vulnerable medial temporal regions provide an additional anatomical rationale for studying olfaction across the AD continuum [5].

Odor-identification impairment is well documented in MCI and AD, and longitudinal studies show that poorer performance can precede cognitive decline or conversion to AD dementia [2,6,7,8]. These findings support olfactory testing as a useful risk-enrichment tool, although not as a disease-specific diagnostic measure.

Meta-analytic evidence reinforces this association. A 2024 meta-analysis of 65 MCI studies and 61 AD studies found larger deficits for odor identification than for odor threshold or discrimination, with moderate impairment in MCI and moderate-to-large impairment in AD [6]. Odor identification also depends on attention, semantic memory, retrieval, naming, and medial temporal–cortical networks, so poor performance can reflect dysfunction at several levels of the olfactory system [5,6,9].

Neuropathological studies provide a complementary rationale. Tau pathology can involve the olfactory bulb and nerve from relatively early disease stages, and amyloid-β and paired-helical-filament tau have also been identified in the human olfactory epithelium [10,11,12,13,14]. Anatomically, olfactory sensory neurons in the olfactory epithelium extend their axons through the cribriform plate and synapse within olfactory-bulb glomeruli. Mitral and tufted cells subsequently convey olfactory information through partly distinct projection patterns to central olfactory and limbic structures, including the anterior olfactory nucleus, olfactory tubercle, piriform cortex, amygdalar regions, and lateral entorhinal cortex [15,16]. This organization represents a well-established feature of the mammalian olfactory system rather than a finding specific to any individual Alzheimer’s disease study. Its relevance to AD is nevertheless notable because the entorhinal cortex is among the medial temporal regions affected early in the disease continuum [5].

Importantly, anatomical communication between the nasal cavity and the central nervous system is not restricted to the olfactory neuroepithelium. The respiratory nasal mucosa is extensively supplied by trigeminal sensory afferents, principally through branches of the ophthalmic (V1) and maxillary (V2) divisions of the trigeminal nerve, and human physiological studies demonstrate trigeminal responsiveness across multiple regions of the respiratory mucosa [17]. Experimental tracing has shown that nasal trigeminal afferents project centrally to the trigeminal brainstem nuclear complex [18]. Moreover, preclinical intranasal-delivery studies demonstrate that molecules can reach CNS structures along trigeminal-associated as well as olfactory-associated pathways, with the trigeminal route particularly associated with access toward brainstem regions [19,20]. Contemporary analyses therefore regard the olfactory and trigeminal systems as complementary anatomical routes relevant to nose-to-brain transport [21]. However, evidence for experimental intranasal transport should not be interpreted as demonstrating that endogenous amyloid-β, tau, immune mediators, or other AD-related molecules naturally propagate from the human nasal mucosa to the brain through these pathways.

The olfactory epithelium adds a distinct experimental opportunity because it can be accessed endoscopically and contains mature and immature sensory neurons, basal progenitors, sustentacular and microvillar cells, and local immune populations [3,4,22]. Recent brush-based single-cell work has shown that such samples can capture disease-associated neuronal and immune states in living, biomarker-characterized participants across preclinical and clinical AD stages [23]. The detailed findings and limitations of that study are considered in Section 3.4 rather than repeated here.

This shift from behavioral testing to tissue biology does not remove the problem of specificity. Aging, chronic rhinosinusitis, smoking, traumatic injury, medication exposure, viral infection, Parkinson’s disease, Lewy body disease, and other conditions can impair olfaction or alter the olfactory mucosa [1,24,25,26]. Persistent post-viral smell loss can include immune-cell infiltration and altered epithelial gene expression, while aging changes olfactory stem-cell states [25,26]. These influences must be separated from AD-related biology in any tissue-based study.

Accordingly, olfactory-cleft sampling should be viewed as an emerging mechanistic platform rather than an established diagnostic test. Its value will depend on whether cell-resolved tissue signatures provide information beyond plasma, CSF, PET, genetic, cognitive, and olfactory measures and whether they predict progression or treatment response in longitudinal cohorts [23,27].

### Literature Search and Selection Strategy

This article was designed as a narrative review aimed at integrating the emerging literature on the olfactory epithelium and olfactory cleft as an accessible tissue compartment for investigating AD, rather than as a systematic review or meta-analysis. The relevant literature was identified through targeted searches of PubMed/MEDLINE, Scopus, Web of Science, and Google Scholar, supplemented by examination of the reference lists of relevant primary studies and reviews. The literature search was updated through August 2026. Search terms were used individually and in combination and included “Alzheimer’s disease”, “preclinical Alzheimer’s disease”, “olfactory epithelium”, “olfactory mucosa”, “olfactory cleft”, “olfactory biopsy”, “nasal biopsy”, “olfactory sensory neurons”, “olfactory neuroblasts”, “olfactory stem cells”, “single-cell RNA sequencing”, “neuroinflammation”, “immune cells”, “CD8 T cells”, “amyloid”, “tau”, and “olfactory dysfunction”.

Priority was given to original human studies directly examining olfactory mucosa, olfactory epithelium, olfactory-cleft samples, or cells derived from these tissues in participants with AD, mild cognitive impairment, biomarker-defined preclinical AD, or appropriate comparison groups. These studies form the principal evidence base of the review and are summarized in Table 1. Historical histopathological studies, patient-derived cellular models, transcriptomic and metabolic investigations, and studies using fresh olfactory tissue were included to illustrate the methodological development of the field. Studies of psychophysical olfactory function, established AD biomarkers, cerebrospinal-fluid drainage, mucosal immunity, aging, post-viral olfactory dysfunction, and relevant animal models were included selectively when they provided mechanistic or clinical context necessary for interpreting the human olfactory-tissue findings. Studies concerned exclusively with olfactory performance without relevance to tissue biology or early AD mechanisms were not reviewed comprehensively.

Because this is a narrative rather than systematic review, study selection was guided by relevance to the biological and methodological questions addressed in the article, and no formal risk-of-bias assessment or quantitative evidence synthesis was performed. Particular attention is given to the study by D’Anniballe et al. [23] because, among the currently available studies, it represents a methodological transition from postmortem tissue and expanded patient-derived cultures to endoscopically obtained fresh olfactory-cleft samples analyzed at single-cell resolution in biomarker-characterized individuals spanning preclinical and clinical AD. Its prominence therefore reflects its direct relevance to the central concept of this review rather than preferential study selection. Importantly, its findings are interpreted within the broader body of olfactory-tissue research, and its small cross-sectional sample, absence of external classifier validation, variable tissue composition, and unresolved disease specificity are explicitly considered when drawing conclusions.

## 2. From Olfactory Testing to Olfactory Tissue Biology

For much of its history, olfactory research in AD was dominated by psychophysical testing of odor detection, discrimination, recognition, and identification. Odor identification received particular attention because it is consistently impaired in MCI and AD, is inexpensive to administer, and depends on higher-order processes including attention, semantic memory, naming, and medial temporal–limbic circuitry [2,7,39].

Longitudinal studies established that poor odor identification can precede dementia. Roberts et al. linked lower Brief Smell Identification Test scores with incident amnestic MCI and progression from amnestic MCI to AD dementia, while Devanand et al. showed that olfactory deficits in MCI predicted later AD [7,39]. These observations established smell testing as a candidate risk marker rather than merely a symptom of established dementia.

The limitation is biological nonspecificity. Odor-identification scores can be affected by aging, sinonasal inflammation, head trauma, smoking, medication, previous viral infection, depression, vascular disease, and other neurodegenerative disorders [39,40]. Persistent post-viral olfactory dysfunction is particularly relevant because inflammatory and transcriptional abnormalities can persist after infection [25]. Behavioral testing therefore cannot identify which peripheral or central mechanism is responsible for a low score.

This limitation shifted attention toward the olfactory mucosa itself. The tissue contains sensory neurons, basal progenitors, sustentacular and microvillar cells, olfactory ensheathing and stromal cells, vascular elements, and resident or recruited immune populations [3,22]. Because sensory axons traverse the cribriform plate to the olfactory bulb, the mucosa occupies an unusual position between the external environment and the central olfactory pathway.

The neuroepithelium can be sampled in living individuals by endoscopic biopsy or, more recently, by cytology brushing [3,41]. However, olfactory epithelium is patchily distributed and becomes more discontinuous with age; adjacent samples may therefore differ markedly in the proportions of olfactory and respiratory epithelium [31,41]. Anatomical targeting and confirmation of olfactory cell-lineage markers are essential for interpretation.

An additional source of heterogeneity is the sampling method itself. The terms “olfactory biopsy” or “olfactory sampling” encompass biologically distinct procedures that should not be treated as interchangeable. Endoscopic tissue biopsy preserves tissue architecture and can permit histological localization, whereas cytology brushing preferentially recovers dissociated superficial cells suitable for cellular and molecular profiling. Biopsy-derived cultures introduce a further level of selection because cell expansion and culture conditions can alter cellular composition, transcriptional states, metabolism, and stress responses relative to freshly obtained tissue. Exfoliated mucosal material represents a compositionally mixed sample, while nasal secretions contain soluble material that may originate from respiratory and olfactory epithelium, glands, inflammatory cells, vasculature, and mucociliary processes rather than from olfactory neuroepithelium specifically. These methodological distinctions are particularly important because human olfactory epithelium is distributed discontinuously within the nasal cavity and may be interspersed with respiratory epithelium; consequently, samples obtained from adjacent sites can differ substantially in cellular composition. Comparisons across studies should therefore consider sampling location, collection technique, fresh versus cultured material, verification of olfactory-cell identity, and the degree of respiratory epithelial contamination. Standardization and transparent reporting of these variables will be essential for determining which findings are reproducible across sampling platforms and which are specific to a particular specimen type.

Early tissue studies were primarily histopathological. Talamo et al. described abnormal olfactory-neuron morphology and immunoreactivity in AD, Tabaton et al. reported tau-reactive neurites in olfactory-mucosal biopsies, and Yamagishi et al. observed tau-related and plaque-like immunoreactivity [28,29,30]. Arnold et al. later detected amyloid-β and paired-helical-filament tau in a substantial proportion of neuropathologically verified AD olfactory epithelia, with severity related to cerebral pathological burden [14].

These findings were not uniform. Godoy et al., studying 12 AD and 24 control cadavers, found that amyloid-β and tau signals varied by nasal sampling site and did not provide clear diagnostic separation [31]. Together, the early studies established that AD-associated pathology can be detected in some olfactory specimens, but also highlighted major anatomical, interindividual, and disease-stage heterogeneity [14,28,29,30,31].

Importantly, several early studies directly challenged the disease specificity and diagnostic sensitivity of olfactory-mucosal pathology. Trojanowski et al. found dystrophic neurites in the olfactory epithelium of all 11 AD cases examined, but similar abnormalities were also present in 11 of 14 individuals with other neurodegenerative disorders and in 6 of 8 neurologically normal adult controls, indicating that this phenotype was not specific to AD [42]. Likewise, Crino et al. detected amyloid-β immunoreactivity in 10 of 12 AD cases, but also in 3 of 10 neurologically normal adult controls, as well as in several cases with other neurological conditions; amyloid-related staining was additionally observed in fetal olfactory tissue, leading the authors to conclude that such deposition can occur under a variety of circumstances and is not restricted to AD [43]. Conversely, Hock et al. found no detectable tau or amyloid-β immunoreactivity in olfactory-mucosal biopsies from five patients with clinically mild-to-moderate AD, although tau immunoreactivity was detected in postmortem olfactory tissue from patients with severe neuropathological disease [44]. These negative and non-specific findings complement later evidence of substantial regional heterogeneity in olfactory amyloid-β and tau pathology and indicate that pathological changes in the olfactory mucosa may vary with disease stage, sampling location, and tissue characteristics rather than representing an invariant AD-specific signature. Consequently, the current literature supports olfactory tissue primarily as an exploratory mechanistic and biological sampling platform, while its sensitivity, specificity, and diagnostic or prognostic utility remain insufficiently established for clinical application.

The transition from static histopathology to living-cell analysis began with primary olfactory-neuroblast cultures. Wolozin et al. reported altered amyloid-precursor-protein processing, Johnson et al. identified reproducible protein-expression differences, and Ghanbari et al. found increased oxidative-damage markers in AD-derived olfactory cells [32,33,34]. These small studies introduced the important concept that olfactory biopsy-derived cells may retain donor-associated molecular phenotypes after isolation and can be used for functional experiments.

Subsequent work extended this approach to amyloid-related phenotypes, non-coding RNA, cellular migration, and other candidate biomarkers. Although several studies reported potentially discriminative molecular signals, most used small clinically defined cohorts, lacked contemporary amyloid or tau confirmation, and had no independent validation. Their main contribution was therefore methodological: demonstrating that living olfactory-derived cells can be collected, expanded, and experimentally manipulated rather than establishing a diagnostic assay.

More recent culture models have enabled transcriptomic and functional analyses. Shahbaz et al. established three-dimensional air–liquid interface cultures from control and mild-AD olfactory mucosa and observed diagnosis-associated differences in responses to SARS-CoV-2 infection [35]. Lampinen et al. combined single-cell RNA sequencing with amyloid and mitochondrial assays, identifying 240 AD-associated differentially expressed genes together with altered respiration and reduced ATP production in AD-derived cultures [36].

A complementary investigation from the same research programme examined biometal homeostasis. AD-derived olfactory-mucosal cells contained increased concentrations of zinc, calcium, and sodium relative to control-derived cells. Transcriptomic analysis identified changes in 17 genes associated with metal-ion binding or metal-related functions, while α2-macroglobulin was increased at both messenger-RNA and protein levels [37]. These results are relevant because disturbances in calcium signalling, zinc handling, proteostasis, and oxidative metabolism are established components of AD pathobiology [45,46,47,48]. Olfactory-derived cells could therefore provide an experimentally tractable system in which relationships among biometal regulation, mitochondrial function, oxidative stress, and amyloid processing can be manipulated. Whether these cellular abnormalities predict cerebral pathology or clinical progression remains unknown. Rantanen et al. identified stage-related transcriptomic differences in neurosphere-derived cells from six AD, six MCI, and six control participants, including changes involving AKAP6 and pathways related to signalling and gene regulation [38]. These culture studies support patient-specific modelling but do not directly reproduce the cellular composition or immune context of fresh olfactory epithelium.

Olfactory-derived cells have also been used for pharmacological and genetically anchored proof-of-concept experiments, illustrating the broader potential of the tissue for perturbation studies. Such models remain exploratory because culture expansion can select particular cell states and modify transcriptional or metabolic phenotypes. The strongest designs will pair fresh, uncultured sampling with functional studies in cells obtained from the same participant.

Animal models provide complementary mechanistic evidence for early involvement of the olfactory system in AD-related pathology, although the interpretation of these findings depends strongly on the model and the molecular pathology being reproduced. In amyloid-focused models, abnormalities of peripheral and central olfactory structures frequently precede more widespread cognitive pathology. In Tg2576 mice, Wesson et al. detected olfactory deficits from approximately 3 months of age together with non-fibrillar Aβ accumulation in the olfactory bulb before deposition in other brain regions [49]. Experimental restriction of mutant APP or Aβ expression to olfactory sensory neurons has further shown that amyloidogenic signaling can disrupt olfactory sensory-neuron survival and axonal targeting even in the absence of extracellular plaques [50,51]. Particularly relevant to the olfactory epithelium, Wu et al. reported a spatial–temporal sequence in APP/PS1 mice in which soluble Aβ was detected in the olfactory epithelium at 1–2 months, subsequently in the olfactory bulb at 3–4 months, and later in the anterior olfactory nucleus, piriform and entorhinal cortices, and hippocampus; early olfactory epithelial pathology coincided with reduced olfactory marker protein and impaired odor detection [52]. This sequence should not, however, be interpreted as direct evidence of transneuronal propagation. Consistent with peripheral olfactory involvement, Tg2576 mice exhibit early BACE1 and Aβ-oligomer abnormalities together with loss of olfactory sensory neurons and thinning of affected regions of the olfactory epithelium [53], whereas 5xFAD mice show region-specific Aβ accumulation, impaired neuronal turnover, and reduced physiological odor responses in olfactory epithelial zones projecting to corresponding olfactory-bulb glomeruli [54]. Manipulation of this pathway can also modify central amyloid pathology: unilateral chemical ablation of the olfactory epithelium in APP/PS1 mice, resulting in olfactory-bulb denervation, reduced Aβ plaque burden in the ipsilateral bulb and also altered plaque load in the neocortex and hippocampus, indicating that olfactory afferent activity and connectivity can influence amyloid deposition [55].

Models incorporating tau pathology provide a complementary line of evidence. In 3xTg-AD mice, which develop both amyloid- and tau-related abnormalities, olfactory impairment and Aβ deposition in the olfactory bulb occur relatively early [56], and more recent work demonstrated significant Aβ and tau pathology, neuroinflammatory activation, and reduced neuronal activity in the olfactory bulb at 4 months, when learning and memory deficits and comparable hippocampal pathology were not yet evident [57]. In P301S tau-transgenic mice, early tauopathy and neurodegeneration in the olfactory bulb and piriform cortex are accompanied by impaired olfactory sensitivity [58], while electrophysiological studies have identified early mitral-cell dysfunction and abnormal olfactory-bulb network oscillations before later cognitive or hippocampal synaptic abnormalities [59,60]. Dourte et al. are particularly relevant to the present biopsy-focused discussion because their experiments directly interrogated the olfactory epithelium rather than restricting analysis to the olfactory bulb or higher olfactory structures. In PS19 mice, phosphorylated tau was detectable in the olfactory epithelium and olfactory nerve layer of the bulb from 1.5 months of age, with later accumulation in connected regions including the piriform and entorhinal cortices and hippocampal formation. Moreover, experimentally stripping the olfactory epithelium reduced downstream pretangle- and tangle-like tau pathology in the piriform cortex, amygdala, and entorhinal cortex [61]. Notably, olfactory discrimination and detection thresholds remained largely preserved in PS19 mice despite this pathology, further demonstrating that pathological involvement and measurable olfactory dysfunction need not progress in parallel.

More recent models broaden this evidence beyond classical familial-AD and MAPT-overexpression paradigms. In App^NL-G-F mice, Meyer et al. identified an early and relatively selective loss of locus-coeruleus noradrenergic axons in the olfactory bulb, accompanied by olfactory impairment and microglial phagocytosis of these axons before substantial amyloid-plaque deposition; genetic attenuation of microglial phagocytosis preserved both noradrenergic innervation and olfactory function [62]. In addition, mice combining humanized APOEε4 with the AD-associated TREM2 R47H variant exhibited age-dependent deficits in odor-guided behavior and altered odor-evoked olfactory-bulb neural and hemodynamic responses, providing evidence for olfactory-system dysfunction in a model designed around genetic risk for late-onset rather than familial AD [63]. Nevertheless, none of these models should be regarded as a direct reproduction of sporadic human AD. APP/PS1, Tg2576, 5xFAD, and 3xTg-AD mice carry disease-associated transgenes, while PS19 mice overexpress mutant human P301S tau and represent a tauopathy model without the amyloid component that defines AD. Accordingly, the animal literature supports the concepts of early olfactory-system vulnerability and anatomically linked interactions between peripheral olfactory pathology and connected brain regions, but it does not establish that sporadic human AD originates in the olfactory epithelium or spreads unidirectionally from the nose to the brain. Against this experimental background, the principal translational significance of the recent D’Anniballe et al. study is that it moves the question back into living, biomarker-characterized humans by determining whether disease-associated neuronal and immune states can be detected directly in freshly sampled olfactory neuroepithelium.

A major methodological transition came with the 2026 D’Anniballe et al. study, which applied endoscopically guided olfactory-cleft brushing and single-cell RNA sequencing to fresh samples from 22 biomarker-characterized participants. The work demonstrated recovery of neuronal, epithelial, progenitor, and immune populations and identified coordinated neural–immune alterations across preclinical and clinical AD [23]. Because this study represents the most direct application to date of fresh, cell-resolved olfactory-cleft sampling across biomarker-defined stages of AD, its quantitative findings and limitations are presented in detail in Section 3.4.

The progression from smell testing to tissue analysis changes the questions that can be addressed. Psychophysical studies ask whether olfactory impairment predicts MCI or dementia, whereas tissue studies can examine which cell populations are altered, whether sensory neurons show metabolic or inflammatory stress, how progenitors respond to injury, and whether immune-cell states change before cognitive symptoms.

These approaches are complementary rather than competing. An informative research framework can combine odor identification with olfactory-cleft sampling, established fluid and imaging biomarkers, genetic risk factors, and longitudinal cognitive assessment, allowing tissue-level mechanisms to be interpreted within the broader AD continuum [27,64].

An adjacent line of research has examined nasal secretions. Kim et al. reported higher nasal amyloid-β-related signals in AD, Yoo et al. described longitudinal relationships between oligomeric Aβ species and clinical progression, and Jung et al. related nasal Aβ42 to amyloid PET burden and cognition across the AD continuum [65,66,67]. These findings are promising but require independent replication.

Nasal-fluid measurements should remain analytically distinct from olfactory-cleft biopsy because secretions can originate from multiple epithelial, glandular, vascular, inflammatory, and mucociliary sources. Pairing nasal fluid with anatomically localized brushing in the same participant could clarify which soluble signals are related to specific neuronal, epithelial, or immune-cell states.

Clinical translation remains premature. Olfactory sampling is more invasive and technically demanding than smell testing or blood collection, tissue yield is anatomically variable, and sinonasal disease, smoking, environmental exposure, allergy, and previous viral injury can independently alter cell states [22,25,31,40,41]. These sources of variability should be handled as predefined methodological factors rather than repeated as caveats throughout the manuscript.

Overall, the field has progressed from behavioral risk markers, through histopathology and patient-derived cultures, to fresh single-cell sampling. Table 1 summarizes the principal human studies and their limitations, including small cohorts, heterogeneous sampling sites, culture-induced selection, incomplete biomarker characterization, and uncertain disease specificity.

## 3. The Olfactory Epithelium as a Neural–Immune Interface

### 3.1. A Specialized Sensory and Immune-Barrier Tissue

The olfactory epithelium is simultaneously a peripheral mucosal barrier and a neural sensory organ. It is exposed to microorganisms, allergens, pollutants, and inflammatory stimuli while containing primary sensory neurons whose axons cross the cribriform plate to the olfactory bulb and connected olfactory and limbic regions [3,4,22]. This combination of environmental exposure, regenerative biology, mucosal immunity, and neural continuity makes the tissue distinctive among accessible human specimens.

The epithelium contains mature and immature sensory neurons, globose and horizontal basal cells, sustentacular and microvillar cells, glands, olfactory ensheathing cells, stromal and vascular elements, and multiple immune populations [3,4,22,68]. Immune surveillance and epithelial repair are normal functions of this niche, but prolonged inflammation can interfere with neuronal regeneration and shift basal-cell programs toward barrier defence [4,26]. The tissue is therefore best regarded as an accessible extracranial neural–immune compartment, not as a substitute for brain tissue.

The term “neuroimmune window” should therefore be interpreted as a conceptual and experimental framework rather than evidence that molecular changes detected in the olfactory mucosa directly reflect neuroinflammation within the CNS. Altered transcriptional, metabolic, or immune-cell states in olfactory-cleft samples may arise from local mucosal inflammation, environmental exposure, aging, infection, systemic immune signals, or disease-associated processes shared between peripheral and central compartments. At present, neither the anatomical proximity of the olfactory mucosa to the brain nor the presence of AD-associated cellular signatures establishes their CNS origin or demonstrates direct correspondence with cerebral neuroinflammatory processes. Establishing such a relationship will require paired analyses of olfactory tissue with CSF, blood, neuroimaging, and, where feasible, brain-derived measures within the same individuals, together with longitudinal and mechanistic studies.

### 3.2. Olfactory Macrophages Are Specialized but Are Not Brain Microglia

Cell-resolved studies require a clear distinction between olfactory-mucosal macrophages and CNS microglia. Shared markers or transcriptional programs can indicate functional convergence but do not establish common developmental identity. Wellford et al. identified long-lived P2RY12-high macrophages positioned near sensory neurons and more inflammatory, antigen-presenting MHC-II-high macrophages in the lamina propria, with related signatures also detected in human biopsy datasets [68].

This heterogeneity means that a myeloid change in AD could reflect altered resident-cell survival, monocyte recruitment, antigen presentation, inflammatory reprogramming, or increased clearance of injured neurons. The 2026 human study used the term microglia-like for some projected transcriptional states; these should be interpreted as mucosal myeloid cells expressing selected microglia-associated programs rather than as parenchymal microglia [23]. Spatial and phenotypic validation will be important in future work.

### 3.3. The Cribriform Plate, Cerebrospinal-Fluid Outflow, and Immune Surveillance

The relationship between the olfactory region and the CNS also involves the cribriform plate, where olfactory nerve bundles, vessels, extracellular spaces, immune cells, and lymphatic structures form a complex border rather than a simple anatomical opening. Experimental studies support CSF outflow and immune surveillance along this route [64,69,70,71,72].

In mice, Spera et al. demonstrated open connections between subarachnoid CSF and lymphatic vessels surrounding olfactory nerves near the cribriform plate [69]. Yoon et al. identified a nasopharyngeal lymphatic plexus that drains toward deep cervical lymphatics and undergoes age-related structural changes [64]. Neuroinflammatory models further show lymphangiogenesis, antigen drainage, and immune-regulatory remodeling in this region [70,71,72].

These data provide a plausible anatomical context for interaction among CNS-derived solutes, peripheral immune surveillance, and the olfactory mucosa, but most causal evidence is from animal models. Anatomical proximity does not demonstrate that human olfactory immune cells have encountered amyloid, tau, or other CNS antigens, nor that a mucosal inflammatory signal originates in the brain rather than from local exposure.

A direct test would require paired sampling across compartments. T-cell receptor sequencing of olfactory tissue, blood, and CSF, combined with antigen-specific functional assays, could determine whether expanded adaptive immune populations are shared between sites or arise independently [73].

### 3.4. Single-Cell Profiling Across the Alzheimer’s Disease Continuum

The strongest direct human evidence for an AD-related neural–immune signature in fresh olfactory tissue comes from D’Anniballe et al. The study included 22 living participants: six cognitively typical biomarker-negative controls, nine cognitively typical participants with an abnormal CSF Aβ42/Aβ40 ratio classified as preclinical AD, and seven cognitively impaired participants with biomarker-supported clinical AD. Endoscopically guided brushing of the superior and posterior olfactory cleft yielded olfactory sensory neurons, progenitors, sustentacular and microvillar cells, T cells, macrophages, dendritic cells, and variable respiratory epithelium for single-cell RNA sequencing and flow cytometry; the integrated dataset contained 220,509 cells [23].

The clearest preclinical immune signal involved memory CD8 T cells. A projected activation program classified approximately 13.4% of olfactory memory CD8 T cells as activation-positive in preclinical AD versus 4.1% in controls. A small flow-cytometric validation, with three participants per group, also showed increased CD38-positive/HLA-DR-positive CD8 T-cell activation. The transcriptional approach inferred an antigen-experienced state but did not identify the antigen, and the small validation sample limits precision [23].

Myeloid and neuronal states also differed across disease stages. Olfactory myeloid cells showed inflammatory and microglia-associated programs, with increased HLA-DQA2 and reduced EGR3 in both preclinical and clinical AD; these cells remain mucosal macrophage or dendritic populations rather than developmentally verified CNS microglia. Olfactory sensory neurons showed parallel stage-related changes, including increased AKR1B1 and decreased CERT1, implicating metabolic, oxidative, lipid-transport, and membrane-homeostasis pathways [23].

Computational ligand–receptor analyses suggested altered neuron–immune communication, including granzyme-A- and macrophage migration inhibitory factor-related interactions. The authors also constructed a combined neuronal-support and immune-expression module that distinguished AD-continuum participants from controls with an area under the receiver-operating-characteristic curve of 0.81 (95% CI 0.68–0.94). The module correlated inversely with CSF Aβ42/Aβ40 but not significantly with CSF p-tau181 or Montreal Cognitive Assessment scores [23].

These findings are discovery-level rather than diagnostic evidence. The classifier was developed and evaluated in the same 22-participant cohort, the study was cross-sectional, and olfactory and CSF single-cell datasets were not prospectively paired within individuals. Consequently, the results show that cell-specific neural and immune abnormalities can be detected in accessible olfactory-region samples from biomarker-characterized participants, but they do not establish AD specificity, causality, prognostic value, or a direct correspondence with intrathecal immunity [23].

### 3.5. Adaptive Immunity and Its Relationship to AD Neurodegeneration

Independent studies provide biological context for the adaptive-immune findings. Gate et al. identified clonally expanded, antigen-experienced CD8-positive effector-memory cells in the blood and CSF of people with AD, including clonotypes shared between compartments [73]. In mouse tauopathy, Chen et al. showed that interactions among activated microglia, infiltrating T cells, and neurodegeneration can be causal, because depletion of microglia or T cells reduced neuronal loss and atrophy [74].

These data make an AD-related interpretation of activated olfactory T-cell states plausible but not proven. Olfactory T cells may reflect central disease, local mucosal injury, microbial or environmental antigens, or systemic inflammatory signals. Establishing mechanism will require paired T-cell receptor sequencing, antigen-specific assays, spatial localization, and longitudinal sampling [23,73,74].

### 3.6. Environmental Exposure and Disease-Dependent Cellular Responses

Because the olfactory mucosa is continuously exposed to inhaled biological and chemical agents, it can be used to study how environmental challenges interact with donor-specific neurodegenerative vulnerability. The same accessibility is also a major source of confounding because local epithelial and immune states can change independently of AD [4].

Patient-derived and postmortem studies illustrate this dual role. Shahbaz et al. found diagnosis-associated differences in transcriptional responses to SARS-CoV-2 in air–liquid interface cultures derived from control and AD olfactory mucosa [35]. Complementary human postmortem evidence was provided by Khan et al., who used rapidly obtained respiratory mucosa, olfactory mucosa, and olfactory-bulb specimens from deceased COVID-19 patients and identified sustentacular cells, rather than olfactory sensory neurons, as the major SARS-CoV-2 target within the olfactory mucosa. No evidence of infection of olfactory sensory neurons or of the olfactory-bulb parenchyma was detected, indicating that virus-associated olfactory dysfunction can arise from disruption of the local epithelial and sustentacular-cell environment without direct neuronal infection or demonstrable propagation into the olfactory bulb [75]. Together, these observations illustrate how viral exposure can substantially modify olfactory-mucosal biology without establishing a causal route from nasal infection to AD. Studies of traffic-related ultrafine particles and diesel emissions similarly reported mitochondrial, redox, inflammatory, transcriptional, and epigenetic responses that differed between AD-derived and control olfactory cells [76,77].

Rössner et al. extended this approach to real-world ambient-air exposure, identifying exposure-related molecular changes in both healthy and AD-derived olfactory-mucosal cells, with relatively stronger inflammatory enrichment in the AD group [78]. Collectively, these studies support the mucosa as a human exposure–response model but do not show that infection or pollution initiates sporadic AD.

Interpretation is limited by donor selection, medication and systemic disease, culture adaptation, exposure dose, cell composition, and the absence of intact vascular and immune systems. Fresh-tissue studies are needed to establish how closely experimental responses reproduce the in vivo olfactory epithelium [35,76,77,78].

### 3.7. Aging, Regeneration, and Major Biological Confounders

Aging is both the strongest risk factor for sporadic AD and a major determinant of olfactory epithelial structure. With age, the neuroepithelium becomes more discontinuous and may be replaced locally by respiratory-like epithelium, so adjacent samples can differ substantially in neuronal, progenitor, sustentacular, and respiratory cell content [25,26,41].

Oliva et al. linked age-related olfactory loss to transcriptional changes in basal stem cells, including cytokine-response and differentiation programs, and showed that cytokine exposure can increase TP63 expression in culture [26]. Persistent post-COVID-19 smell loss likewise includes sustained T-cell infiltration and altered epithelial gene expression despite no detectable persistent virus in sampled tissue [25]. These findings demonstrate that inflammatory neural-support changes are not specific to AD.

Chronic rhinosinusitis, allergy, smoking, head trauma, medication use, environmental exposure, and other neurodegenerative disorders are additional sources of variation [4,25,40]. Studies should therefore prespecify sinonasal assessment, endoscopy, smoking and exposure histories, medication review, and recent-infection screening rather than treating these factors as incidental clinical characteristics.

Sampling itself is another source of heterogeneity. Cell proportions can vary with anatomical targeting and tissue composition, so disease comparisons should emphasize within-cell-type transcriptional analyses, marker-based confirmation of olfactory identity, standardized or bilateral sampling, and transparent reporting of cell yield [23,25,26].

### 3.8. Relationship to Established AD Biomarkers

Olfactory-cleft sampling should be evaluated alongside, not against, established AD biomarkers. CSF Aβ42/Aβ40 and phosphorylated tau, amyloid and tau PET, and validated plasma phosphorylated-tau assays provide increasingly accurate evidence of AD pathology [27,79]. These measures are better suited to detecting canonical disease biology than an experimental nasal biopsy.

The potential niche of olfactory sampling is different: it provides living neuronal, epithelial, progenitor, and immune cells for transcriptional, metabolic, receptor, and functional analyses. Its plausible near-term uses are mechanistic research, biological stratification, and pharmacodynamic studies, provided that endoscopic targeting and tissue quality are standardized. Multimodal studies should test whether these cell-resolved measures add information beyond established biomarkers [23,27,79].

**Figure 1:**
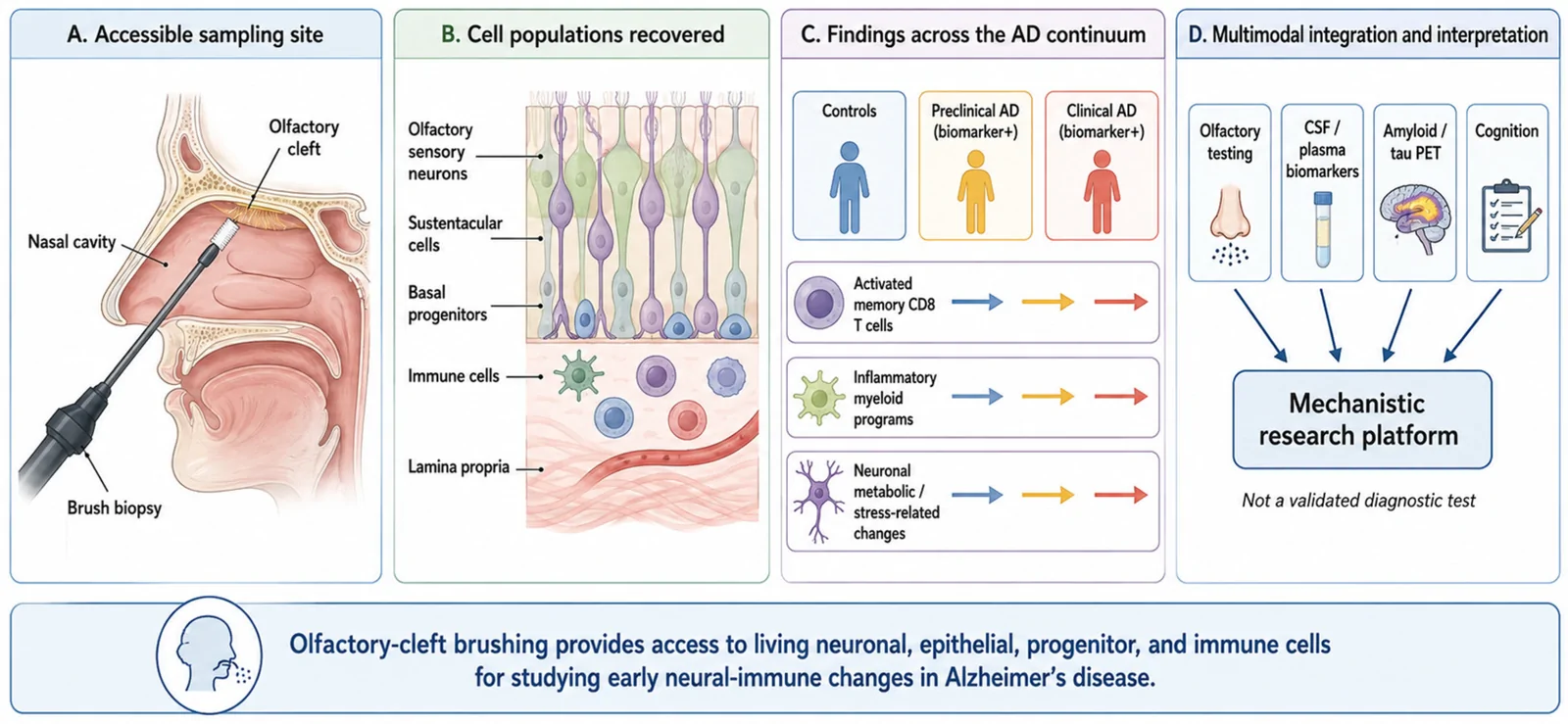
Olfactory-cleft sampling as an accessible neuroimmune research platform in Alzheimer’s disease. Endoscopically guided brushing of the olfactory cleft provides access to neuronal, epithelial, progenitor, and immune-cell populations that can be examined using cell-resolved molecular approaches. Recent single-cell analyses have identified AD-associated changes involving activated memory CD8 T-cell states, inflammatory myeloid programs, and metabolic or stress-related alterations in olfactory sensory neurons across biomarker-characterized participants spanning cognitively unimpaired controls, preclinical AD, and clinical AD. Olfactory-tissue measurements may be integrated with psychophysical olfactory testing, cerebrospinal-fluid and plasma biomarkers, amyloid and tau PET, and longitudinal cognitive assessment. The figure represents a conceptual research framework rather than a validated diagnostic or prognostic model; the direction and magnitude of individual cellular abnormalities should not be interpreted as established monotonic changes across disease stages.

The potential role of olfactory-cleft sampling within a multimodal AD research framework is summarized in Figure 1. Endoscopically obtained olfactory tissue provides access to neuronal, epithelial, progenitor, and immune-cell populations that can be characterized alongside established measures of AD biology and clinical function. Rather than replacing CSF, plasma, PET, cognitive, or olfactory assessments, olfactory-cleft sampling may complement these modalities by providing living cells for cell-resolved investigation of neural–immune and metabolic alterations. Current evidence remains discovery-level, and the framework illustrated in Figure 1.

## 4. Preclinical Alzheimer’s Disease: The Key Opportunity

### 4.1. Why the Preclinical Stage Is the Decisive Setting

The strongest rationale for olfactory-cleft research is the preclinical phase, when AD-related molecular abnormalities are present but objective cognitive impairment has not yet emerged [27,80,81]. Amyloid can accumulate years before dementia, while tau pathology, synaptic dysfunction, glial activation, vascular changes, and neurodegeneration evolve at different rates across individuals [27,82].

Established fluid and imaging biomarkers can identify these processes with increasing accuracy, but they do not provide viable tissue-resident cells for functional analysis. Olfactory-cleft sampling could therefore complement, rather than replace, plasma, CSF, or PET by showing how an accessible neural–immune tissue responds to biologically defined disease [23,27].

### 4.2. Biological Definitions of Preclinical AD

Preclinical AD is defined biologically rather than by symptoms alone. The 2018 NIA–AA Research Framework organized biomarkers into amyloid, tau, and neurodegeneration/neuronal-injury categories, allowing cognitively unimpaired people with abnormal amyloid biomarkers to be placed on the Alzheimer’s pathological continuum [80]. The 2024 Alzheimer’s Association criteria retained this biological orientation while incorporating increasingly accurate blood-based biomarkers [27].

The label preclinical AD should nevertheless be used precisely. Amyloid-positive/tau-negative and amyloid-positive/tau-positive individuals have different biological profiles and progression risks [27,82]. Olfactory studies should therefore report the exact biomarker criteria used for group definition rather than treating all cognitively unimpaired biomarker-positive participants as equivalent.

### 4.3. Behavioral Evidence That Olfactory Abnormalities Emerge Early

Psychophysical evidence provides the clinical rationale for studying olfaction before dementia. In cognitively unimpaired older adults, poorer odor identification has been related to smaller hippocampal volume, thinner entorhinal cortex, worse episodic memory, and, in selected studies, AD biomarker abnormalities [83,84].

Kreisl et al. found that better odor identification strongly predicted the absence of amyloid PET positivity, whereas poor performance alone had limited positive predictive value; both poor olfaction and positive amyloid PET independently predicted later memory decline [85]. This asymmetry illustrates why smell testing may help enrich risk without functioning as an AD-specific test.

Population studies extend this association. Yaffe et al. linked poor odor identification with later dementia risk in 2428 older adults, while Devanand and Roberts and colleagues associated olfactory impairment with cognitive decline, incident MCI, or progression to AD dementia [7,8,39,86].

Neuropathological work also suggests stage-dependent involvement of the olfactory pathway, with tau-related changes becoming increasingly prominent across olfactory structures. These findings support early olfactory dysfunction as a marker of disease vulnerability but do not localize its source uniquely to the epithelium, bulb, medial temporal cortex, or broader cognitive networks.

### 4.4. Subjective Cognitive Decline and Early Biomarker Associations

Subjective cognitive decline is heterogeneous: some individuals have preclinical AD pathology, whereas others report symptoms related to mood, sleep, medical conditions, or normal aging [87]. Olfactory impairment has nevertheless been reported at this stage and becomes more pronounced across MCI and AD dementia [88].

Imaging studies suggest that early olfactory impairment may relate more consistently to tau-related or neurodegenerative processes than to amyloid burden alone. Risacher et al. associated poorer odor identification with greater tau PET signal but not cortical amyloid, and Klein et al. related olfactory impairment to tau pathology and neuroinflammatory PET measures [89,90].

Olfactory-mucosal proteomics offers an intermediate approach between psychophysical testing and cell-resolved biopsy. In 108 adults with subjective cognitive complaints, Sadhukhan et al. identified a broad set of exfoliate proteins associated with memory performance and pathways involving mitochondrial, neurotrophic, complement, extracellular-matrix, and inflammatory biology. Because the cohort was not biomarker-confirmed and the samples were compositionally mixed, these proteins remain exploratory candidates rather than validated AD biomarkers.

Taken together, behavioral and molecular findings support the study of olfaction before dementia but also show that smell loss cannot be equated with amyloid positivity. Direct tissue analysis is potentially useful precisely because it may help separate local neuronal, epithelial, and immune mechanisms from the final behavioral output.

### 4.5. Direct Tissue Evidence and Complementarity with Established Biomarkers

The 2026 single-cell study provides the first direct evidence that fresh olfactory-cleft samples from cognitively unimpaired, amyloid-biomarker-positive participants can contain altered neuronal and immune states [23]. The quantitative findings are summarized in Section 3.4; here, the key implication is narrower: tissue-level abnormalities can be detected before clinically apparent cognitive impairment, but the cross-sectional design does not establish that they precede cerebral pathology or predict progression.

This creates a complementary role for olfactory tissue. Plasma and CSF assays quantify soluble biomarkers, while PET localizes aggregated amyloid or tau; olfactory sampling instead provides viable cells for mechanistic analysis [27]. The discovery cohort was too small for diagnostic validation, so the appropriate question is whether reproducible cell-resolved signatures add mechanistic or prognostic information beyond established biomarkers, not whether they can replace them [23].

### 4.6. Biological Heterogeneity Within Preclinical AD

Cognitively unimpaired amyloid-positive individuals have markedly variable trajectories. In a multicentre study of 1325 participants, Ossenkoppele et al. found substantially greater risk of cognitive decline and progression to MCI in people positive for both amyloid and tau PET than in amyloid-positive/tau-negative individuals [82]. The preclinical population should therefore not be treated as a uniform biological group.

Olfactory tissue may ultimately contribute to this stratification if cellular signatures distinguish stable from progressing biomarker-positive individuals. At present this is a hypothesis: the available single-cell cohort was cross-sectional and did not test prediction of cognitive decline, tau accumulation, neurodegeneration, or conversion to symptomatic disease [23].

### 4.7. Blood Biomarkers and Long-Term Olfactory Decline

Longitudinal blood-biomarker data further connect olfactory decline with early AD-related biology. In 1868 participants followed for up to 15 years, higher baseline p-tau217, p-tau181, neurofilament light, and glial fibrillary acidic protein, together with a lower Aβ42/Aβ40 ratio, were associated with faster subsequent olfactory decline [91].

These findings establish temporal associations between molecular abnormalities and worsening smell function but do not localize the responsible pathology to the olfactory epithelium. Combining longitudinal blood biomarkers, standardized olfactory testing, and cell-resolved tissue sampling could address that mechanistic gap.

### 4.8. Two Complementary Opportunities: Screening and Mechanism

Preclinical olfactory research has two distinct roles. The first is functional risk enrichment, in which inexpensive smell testing identifies people who may warrant more specific biological assessment. The second is mechanistic characterization, in which olfactory-cleft sampling examines neuronal, epithelial, regenerative, and immune processes within biomarker-defined participants [23].

The first role is supported by a larger behavioral literature but has limited disease specificity; the second is biologically richer but remains discovery-level. A sequential multimodal strategy—smell testing for function, established biomarkers for AD status, and tissue sampling for cellular mechanisms—is therefore more defensible than using any one measure in isolation.

### 4.9. Potential Value for Early Therapeutic Studies

Disease-modifying trials are moving toward cognitively unimpaired or minimally impaired participants with biomarker evidence of pathology, as illustrated by prevention studies such as AHEAD 3–45 [92]. This increases the importance of biomarkers that can distinguish molecular target engagement from later clinical benefit.

Repeated olfactory-cleft sampling could, in principle, provide a pharmacodynamic readout of cellular neural–immune responses that are not directly measured by plasma, CSF, or PET. Before such use, studies must establish procedural repeatability, test–retest reliability, sensitivity to treatment, and correspondence with independent measures of target engagement or clinical progression [23,27].

### 4.10. Olfactory–Biomarker Discordance and Disease Specificity

Odor identification is the final output of a distributed system that includes nasal airflow, sensory-neuron function, olfactory-bulb processing, cortical and medial temporal networks, attention, semantic memory, and naming. Dysfunction at any level can reduce performance, so smell loss may reflect central AD pathology, local sinonasal disease, previous viral injury, smoking, vascular lesions, Lewy body pathology, or several interacting causes [25,83,84,93,94,95].

For the same reason, tissue-level immune or neuronal-support abnormalities cannot be assumed to be AD-specific. Persistent post-viral smell loss and chronic rhinosinusitis can produce overlapping inflammatory or epithelial changes [25,95]. The goal is therefore not a stand-alone olfactory diagnosis but identification of cell-resolved signatures that improve biological interpretation when combined with amyloid, tau, neurodegeneration, cognition, genetics, endoscopic findings, and clinical history.

Before olfactory-cleft molecular or cellular signatures can be considered for clinical translation, their disease specificity must be established in appropriately designed comparison cohorts. This is particularly important because aging, persistent post-viral olfactory dysfunction, chronic rhinosinusitis, allergy, smoking, and other inflammatory or environmental exposures can produce epithelial and immune alterations that may overlap with those observed in AD [24,25,40,95]. Neurological disease controls are equally essential. Parkinson’s disease and dementia with Lewy bodies should be prioritized because both are strongly associated with olfactory dysfunction and may involve pathological processes within the olfactory system, creating a particularly relevant differential-diagnostic challenge [24,93]. Future validation studies should therefore compare biomarker-defined AD not only with cognitively healthy age-matched controls but also with Parkinson’s disease, dementia with Lewy bodies, chronic rhinosinusitis, and persistent post-viral olfactory dysfunction using standardized sampling and cell-resolved analyses. Demonstrating that candidate neuronal, epithelial, or immune signatures distinguish AD from these biologically plausible alternative causes of olfactory-tissue alteration is a prerequisite for claiming disease specificity or considering olfactory-cleft sampling for diagnostic, prognostic, or treatment-monitoring applications.

### 4.11. Priorities for Longitudinal Validation

Future studies should stratify cognitively unimpaired participants by explicit biological profiles, including amyloid-negative/tau-negative, amyloid-positive/tau-negative, and amyloid-positive/tau-positive groups, with additional consideration of quantitative tau burden, plasma p-tau217, APOE genotype, subjective cognitive decline, and objective olfactory impairment [27,82].

Olfactory-cleft sampling should be anatomically standardized and paired with marker-based verification of tissue composition. Candidate measurements may include T-cell activation and clonality, myeloid states, sensory-neuron stress, progenitor differentiation, epithelial-barrier programs, and cell–cell communication, interpreted alongside odor testing, plasma and CSF biomarkers, amyloid and tau PET, MRI, and cognition [23].

The critical test is longitudinal prediction. Repeated sampling should determine whether tissue signatures forecast MCI conversion, cognitive decline, tau progression, neurodegeneration, or worsening olfactory function beyond established predictors such as amyloid status, tau burden, age, APOE genotype, and baseline cognition. Internal classification within a discovery cohort is not sufficient evidence of prognostic utility.

Multicentre studies will also need harmonized brush type and anatomical targeting, processing intervals, cell-quality thresholds, sinonasal assessments, medication and exposure histories, recent-infection screening, and estimates of within-person and bilateral variability. These design features are more important for validation than repeatedly restating the findings of a single discovery cohort.

## 5. Conclusions

Olfactory-cleft sampling extends AD olfactory research beyond behavioral smell testing by providing living neuronal, epithelial, progenitor, and immune cells for molecular analysis. Histopathological and culture studies established the biological plausibility of this approach, while recent single-cell work shows that cell-resolved neural–immune abnormalities can be detected in biomarker-characterized individuals, including during cognitively unimpaired stages [23].

The evidence remains preliminary. Existing studies are small, frequently cross-sectional, vulnerable to anatomical and inflammatory confounding, and insufficient to establish AD specificity, causality, prognosis, or diagnostic accuracy. Olfactory-cleft sampling should therefore be treated as a mechanistic research platform that complements established fluid and imaging biomarkers. Its clinical relevance will depend on standardized multicentre and longitudinal studies showing reproducibility, disease specificity, added prognostic value, and sensitivity to treatment response.

## Figures and Tables

**Table 1:** Human olfactory-tissue studies in Alzheimer’s disease.

Study	Participants or Specimens	Olfactory Material	Methods	Principal Findings	Main Limitations and Interpretation
Talamo et al. [28]	Postmortem specimens from individuals with AD	Olfactory mucosa and olfactory neurons	Morphological and immunohistochemical examination	Abnormal morphology, distribution, and immunoreactivity of olfactory neurons suggested that peripheral olfactory neurons may exhibit AD-associated changes.	Postmortem design; limited cohort characterization; findings could not establish diagnostic sensitivity or specificity.
Tabaton et al. [29]	Patients with clinically probable AD	Olfactory-mucosal biopsies	Histopathology, immunohistochemistry, and ultrastructural assessment	Tau-reactive neurites containing abnormal straight filaments resembling filaments identified in AD brain tissue were detected.	Small, clinically defined cohort; predated contemporary biomarker criteria; uncertain anatomical reproducibility and disease specificity.
Yamagishi et al. [30]	Patients with AD and comparison specimens	Olfactory mucosa	Tau and amyloid immunohistochemistry	Neurofibrillary-tangle-like tau immunoreactivity was observed in olfactory receptor-cell dendrites, perikarya, and nerve bundles. Plaque-like extracellular material showed strong tau and weaker amyloid immunoreactivity.	Small cohort; heterogeneous sampling; uncertain reproducibility; findings did not establish a reliable biopsy-based diagnostic test.
Arnold et al. [14]	Neuropathologically verified AD and control cases	Postmortem olfactory epithelium	Amyloid-β and paired-helical-filament tau immunohistochemistry	Amyloid-β and tau pathology was detected in a substantial proportion of AD specimens. Olfactory epithelial pathology was associated with cerebral pathological burden.	Pathology was not uniformly distributed; a negative sample could not exclude AD; postmortem tissue may primarily represent advanced disease.
Godoy et al. [31]	12 AD cadavers and 24 controls	Postmortem olfactory mucosa sampled from different nasal regions	Amyloid-β and paired-helical-filament tau immunohistochemistry	Pathological signals varied by anatomical sampling site, and separation between AD and control specimens was incomplete.	Demonstrated major regional heterogeneity; findings did not support a simple positive-or-negative olfactory biopsy test for AD.
Wolozin et al. [32]	Individuals with sporadic AD and controls	Biopsy-derived primary olfactory neuroblast cultures	Cell culture and amyloid precursor protein analysis	AD-derived cultures showed abnormal amyloid precursor protein processing, including increased abundance of an amyloid-β-containing precursor.	Small clinically diagnosed cohort; culture conditions may select particular cell populations and alter their phenotype.
Johnson et al. [33]	AD-derived and control olfactory neuroblast cultures	Biopsy-derived cultured olfactory cells	Two-dimensional gel electrophoresis and protein-expression analysis	Five reproducible protein-expression differences were identified between AD-derived and control cultures.	Exploratory proteomic analysis; small sample; uncertain relationship between cultured-cell changes and intact olfactory tissue in vivo.
Ghanbari et al. [34]	Cultures obtained from patients with AD and controls	Cultured olfactory neurons	Immunochemical analysis of oxidative-damage markers	AD-derived cells showed increased 4-hydroxynonenal, Nε-carboxymethyllysine, and heme oxygenase-1 immunoreactivity. Modified proteins accumulated in lysosome-like structures in one AD-derived culture.	Small sample; oxidative abnormalities are not specific to AD; culture adaptation may influence cellular stress responses.
Shahbaz et al. [35]	Three cognitively healthy donors and three individuals with mild AD dementia	Olfactory-mucosal biopsy-derived cells	Three-dimensional air–liquid interface culture, SARS-CoV-2 exposure, and transcriptomic analysis	Demonstrated that living olfactory-mucosal cells can be collected, expanded, differentiated, and experimentally challenged. AD-derived cultures showed altered oxidative, inflammatory, antiviral, and olfactory-related responses.	Designed as an infection-response model rather than an AD biomarker study; very small cohort; cultured tissue did not reproduce the complete native immune and vascular environment.
Lampinen et al. [36]	Patients with AD and cognitively healthy controls	Cultured olfactory-mucosal cells	Amyloid-β measurement, single-cell RNA sequencing, and mitochondrial functional assays	AD-derived cultures secreted more amyloid-β. Five main cell populations and 240 AD-associated differentially expressed genes were identified. Mitochondrial respiration and ATP production were reduced.	Cultures were dominated by basal-cell-like, fibroblast-like, stromal, and myofibroblast-like populations rather than mature olfactory neurons; culture expansion may alter transcriptional and metabolic states.
Lampinen et al. [37]	AD-derived and control olfactory-mucosal cell cultures	Cultured olfactory-mucosal cells	Elemental measurements, transcriptomics, and protein analysis	AD-derived cells contained higher zinc, calcium, and sodium concentrations. Seventeen metal-related genes were altered, and α2-macroglobulin was increased at transcript and protein levels.	Findings demonstrate an experimental model of biometal dysregulation but do not establish that these abnormalities predict brain pathology or clinical progression.
Rantanen et al. [38]	Six participants with AD, six with MCI, and six cognitively healthy controls	Olfactory neurosphere-derived cells	Cell expansion and transcriptomic analysis	Identified 225 differentially expressed genes between AD and controls, 431 between AD and MCI, and 209 between MCI and controls. Alterations involved AKAP6, vitamin D receptor signaling, MMP2, alternative splicing, sex, and APOE-related effects.	Small exploratory groups; greatest differences occurred between AD and MCI rather than AD and controls; cellular states may reflect both donor biology and culture conditions.
D’Anniballe et al. [23]	Six biomarker-negative controls, nine cognitively unimpaired participants with abnormal CSF amyloid biomarkers, and seven participants with biomarker-confirmed clinical AD	Fresh endoscopically guided olfactory-cleft cytology-brush samples	Single-cell RNA sequencing, computational cell-state and ligand–receptor analyses, and flow cytometry	Generated a dataset of 220,509 cells. Activated memory CD8 T-cell states were prominent in preclinical AD. Myeloid inflammatory programs and olfactory-neuron metabolic and stress-related alterations were detected. A combined neuronal–immune module distinguished the AD-continuum groups from controls with an AUC of 0.81 and correlated with CSF Aβ42/Aβ40.	Small cross-sectional discovery cohort; classifier lacked external validation; flow-cytometric validation included only three participants per group; variable respiratory epithelial contamination; AD specificity remains unestablished.

## Data Availability

No new data were created or analyzed in this study. Data sharing is not applicable to this article.
